# Non-alcoholic fatty liver disease and its associations among adolescents in an urban, Sri Lankan community

**DOI:** 10.1186/s12876-017-0677-7

**Published:** 2017-11-29

**Authors:** Shaman Rajindrajith, Arunasalam Pathmeswaran, Chamilka Jayasinghe, Dulani Kottahachchi, Anuradhani Kasturiratne, Shamila T. de Silva, Madunil A. Niriella, Anuradha S. Dassanayake, Arjuna P. de Silva, H. Janaka de Silva

**Affiliations:** 1 Department of Paediatrics, Faculty of Medicine, University of Kelaniya, Ragama, 11010 Sri Lanka; 2 Department of Public Health, Faculty of Medicine, University of Kelaniya, Ragama, 11010 Sri Lanka; 3 Department of Physiology, Faculty of Medicine, University of Kelaniya, Ragama, 11010 Sri Lanka; 4 Department of Medicine, Faculty of Medicine, University of Kelaniya, Ragama, 11010 Sri Lanka; 5 Department of Pharmacology, Faculty of Medicine, University of Kelaniya, Ragama, 11010 Sri Lanka

**Keywords:** Fatty liver disease, Risk factors, Metabolic syndrome, Obesity

## Abstract

**Background:**

Nonalcoholic fatty liver disease (NAFLD) is a common problem across the world. We aimed to determine the prevalence of NAFLD and its associations in Sri Lankan adolescents living in an urban Sri Lankan community*.*

**Method:**

The study population consisted of the birth cohort of the year 2000, residing in the Ragama Medical Officer of Health area. Socio-demographic and anthropometric data [anthropometric measurements, blood pressure and total body fat distribution] of these adolescents were collected by trained data collectors. Fasting blood sugar, serum insulin, fasting serum lipids and serum alanine aminotransferase (ALT) levels were measured and an abdominal ultrasound was performed. NAFLD was diagnosed on established ultrasound criteria for fatty liver and absent alcohol consumption.

**Results:**

The study sample consisted of 499 adolescents [263 (51.8%) girls]. Forty two (8.4%) had NAFLD. NAFLD was significantly associated with being breast fed for less than 4 months (33.3% vs. 17.1 in controls, *p* = 0.02), higher waist circumference (prevalence risk ratio 83.3/20.3, 4.1, *p* < 0.0001), higher body mass index (prevalence risk ratio 40.5/4.8, 8.4, *p* < 0/0001),higher HOMA-IR (3.7 vs. 1.9, *p* < 0.0001) and high triglycerides (prevalence risk ratio 14.3/5.8, 2.5, *p* = 0.033). Adolescents with NAFLD also had a higher amount of total body fat (*p* < 0.001) and subcutaneous fat (*p* < 0.001) than those without NAFLD. The number of children with metabolic derangements was higher among adolescents with NAFLD than those without (85.8 vs 26.3 in controls, *p* < 0.0001), but a family history of hypertension, diabetes, myocardial infarction or dyslipidaemia were not.

**Conclusion:**

Prevalence of NAFLD was high in Sri Lankan adolescents, and was associated with metabolic derangements, especially obesity, insulin resistance and early cessation of breast feeding.

## Background

Non-alcoholic fatty liver disease (NAFLD) and Metabolic Syndrome (MetS) are two interrelated major global public health problems. NAFLD is considered the commonest liver disease in children and may cause significant liver damage, and leading to cirrhosis in some of them [1, 2]. MetS is a clustering of obesity, atherogenic dyslipidaemia, hypertension and insulin resistance, with the potential for increased risk of serious long term consequences [3].

The burden of NAFLD in adolescents has not been extensively studied, especially in South Asia. A recent systematic review and meta-analysis of epidemiological studies of NAFLD in children reported a pooled mean prevalence of 7.6% in population based studies and 34.2% in studies based in child obesity clinics [4]. Evaluation of National Health and Examination survey USA, between 1988 and 1994 and 2007–2010 showed prevalence of NAFLD rising from 3.9% in 88–94 to 10.7% in 2007–2010. In addition, during 2007–2010, nearly half (48%) of obese males had NAFLD [5]. Although not as well defined as in adults, the prevalence of MetS in adolescents has risen from 4% to 9% from 1988 to 1994 to 2001–2006 in the USA, and for those who were obese, the prevalence varied from 30% to 50% [4, 6]. In 2013, Wickramasinghe and co-workers found that the prevalence of MetS in school children in Colombo, Sri Lanka, was 1.6% [7].

There is strong association between NAFLD and components of MetS. A case control study found that obese children with NAFLD had a significantly higher risk of developing MetS than obese controls without evidence of NAFLD [8]. Pacifico et al. reported that 65.8% children with biopsy proven NAFLD had MetS [9], and Patton et al. studied 254 children with biopsy proven NAFLD and found that the risk of MetS was greater in those with severe steatosis, hepatocellular ballooning, and advanced hepatic fibrosis [10]. A Japanese study showed a linear relationship between adolescent NAFLD and components of MetS [11]. Studying 1170 adolescents in the Raine birth cohort, Ayonrinde and co-workers reported a higher prevalence of MetS in 17 year olds with NAFLD. In addition, this study found an association between the severity of hepatic steatosis and body mass index, waist circumference, subcutaneous adipose tissue thickness and insulin resistance [12]. It has also been shown that arterial stiffness in the presence of NAFLD is related to the deranged metabolic profile [13]. Studies in adults have shown a much clearer association between NAFLD and MetS [14].

Most of the studies on NAFLD in adolescents are from Western countries and the epidemiological patterns, risk factors and associations of NAFLD have not been systematically studied in low and middle income countries, where there is a dual burden of malnutrition and emerging over nutrition.

The objectives of this study were to determine the prevalence of NAFLD, identify possible risk factors and investigate associations between NAFLD and components of MetS in a cohort of adolescents living in an urban area in Sri Lanka.

## Method

### Study design, population and location

This cross sectional study was conducted in the Ragama Medical Officer of Health (MOH) administrative area situated 18 km north of the capital, Colombo. The study was part of a large community-based investigation on non-communicable diseases: the Ragama Health Study (RHS). The RHS is a collaborative study between the International Medical Centre of Japan (IMCJ) and the Faculty of Medicine, University of Kelaniya, Sri Lanka. The first part of this study was conducted in 2007 (on a cohort of 35–64 years olds) [15]. In 2014, a follow up study was performed to re-evaluate the initial cohort. The current study (on adolescents) was planned during the 2014 follow up study. The population has urban characteristics and a multi-ethnic population.

### Population

The study population consisted of children born in the year 2000. When the study was initiated in 2014, they were 14 years old. A sample of 456 adolescents was required to obtain a 95% confidence interval of +/− 2% around a prevalence estimate of 5%. In order to achieve this sample size all adolescents who were born during year 2000 were invited in writing and by telephone to participate in the study, as the annual birth cohort for the Ragama MOH area was about 1000 and the expected response rate was about 50%. Since they were from all parts of the Ragama MOH area, the sample represents the broader population of the area. The purpose of the study, the procedures involved and the potential problems and benefits were explained in detail to both parents and selected children. Written consent from the parents and assent from the adolescents were obtained prior to the recruitment.

### Data collection

All participants were requested to attend a detailed assessment at a special clinic at the Faculty of Medicine, University of Kelaniya, Sri Lanka. They were requested to present to the unit after a 12 h fast. After registration, children and their parents were interviewed by trained interviewers to obtain information on sociodemographic variables, birth history, history and details of breast feeding and life style habits. Child health development records and all available medical records were also checked to rule out other potential medical problems of the cohort. In addition, a detailed history of the parents was also obtained which included checking available medical records.

A complete physical examination was conducted on the adolescents including anthropometric measurements (weight, height and waist circumference). Weight was measured using a digital weighing scale with a graduation of 100 g (Seca 893). The height was measured using a portable stadiometer with a graduation of 1 mm (Seca 213). Waist circumference was measured using a body circumference measuring tape with a graduation of 1 mm (Seca 203) (Seca Deutschland**,** Medizinische Messsysteme und Waagen. Hamburg, Germany). The blood pressure (both systolic and diastolic) was measured 2 times with an interval of 3 min, using an automated blood pressure monitoring system (Omron Healthcare Co LTD Kyoto, Japan). The average of the 2 values for both systolic and diastolic pressures were taken as the final measure. Total body fat (TBF) and visceral fat percentage (VFP) were measured using a body composition monitor using the proven bioelectrical impedance method according to the instruction manual (Omron HBF-362 body composition monitor, Omron Healthcare Co LTD Kyoto, Japan). All subjects underwent ultrasonography of the liver with a 5-MHz 50 mm convex probe (MindrayDP-10 Ultrasound Diagnostic Systems, Mindray Medical International Limited, Shenzhen, China). Ultrasonographic examination was carried out by five doctors who had been specially trained in liver ultrasonography. A 10 mL sample of venous blood was obtained from each subject. This was used to determine fasting serum triglycerides (TG), high density lipoproteins cholesterol (HDL), low density lipoprotein cholesterol (LDL), very low density lipoprotein cholesterol (VLDL), serum alanine aminotransferase activity (ALT), fasting plasma glucose levels and serum insulin.

### Definitions

Fatty liver was diagnosed in the presence of two or three of the following three criteria on ultrasonographic evaluation: increased hepatic echogenicity compared to the spleen or the kidney, blurring of the liver vasculature and deep attenuation of the ultrasonograpic signal [16]. This has an adequate threshold for the detection of steatosis within more than 33% of hepatocytes on liver histology [17]. NAFLD was defined as the presence of fatty liver on ultrasound with safe or absent alcohol consumption. Adolescents who did not have any of the three ultrasonographic features of fatty liver served as non-NAFLD controls. The homeostasis model assessment for insulin resistance (HOMA-IR score was calculated by using the following formula: HOMA-IR score = (fasting insulin [μU/ml]X Fasting glucose [mmol/L])/22.5.

### Statistical analysis

Data were entered to a custom database created in EpiData version 3 (The EpiData Association, Odense, Denmark) and logical and random checks were done to minimize errors in data entry. Statistical analysis was done using Stata Version 14 (StataCorp, College Station, Texas, USA). Continuous and categorical data were summarized using mean and standard deviations and percentages, respectively. Bivariate analysis was done using the two sample t test and Chi squared test as appropriate. *P* < 0.05 was considered as significant.

### Ethical approval

Ethical approval was obtained from the Ethics committee, Faculty of Medicine, University of Kelaniya, Sri Lanka.

## Results

Five hundred and eight [263 (51.8%) girls] participated in the study. Seven were exclude from the analysis as they were out of the age of 14 years by the time of recruitment. Two adolescents refused to undergo ultrasonography and were hence excluded from the study. Four hundred and ninety nine (499) adolescents were included in the final analysis [257 (51.5%) girls]. Of these 499 adolescents, 42 (8.4%; 95% CI 5.9–10.7) had NAFLD: fatty liver on ultrasound and none consumed alcohol. The 373 adolescents who did not have any of the three features of fatty liver on ultrasonography served as non-NAFLD controls. Eighty four (84) adolescents who had one sonographic feature of fatty liver were excluded from the analysis as they could not be considered as either having NAFLD or as true controls Table 1 provides the characteristics of adolescents included in the analysis.

**Table 1:** Characteristics of the sample of adolescents in the study

Variable	Girls (257) Mean (SD)	Boys (242) Mean (SD)	*p* value^a^
Mean height (cm)	154.4 (5.8)	159.5 (7.5)	<0.001
Mean weight (kg)	45.5 (8.5)	46.4 (11.2)	0.032
Mean systolic pressure (mmHG)	102.8 (11.5)	104.3 (12.9)	0.214
Mean diastolic pressure (mmHg)	66.1 (7.5)	63.2 (8.5)	<0.001
Mean BMI (kg/m^2^)	19.0 (3.2)	18.1 (3.8)	0.003
BMI z score for age	−0.5 (1.2)	−0.9 (1.5)	0.001
ALT (IU/L)	12.6 (5.4)	15.1(7.6)	<0.001
Fasting blood glucose (mg/dL)	78.4 (13.5)	80.0 (7.7)	0.10
Serum insulin levels (μU/mL)	11.3 (7.2)	9.9 (6.4)	0.02
Total cholesterol (mg/dL)	169.6 (29.7)	155.0 (24.8)	<0.001
HDL cholesterol (mg/dL)	48.0 (1.4)	47.5 (1.5)	<0.001
LDL cholesterol (mg/dL)	103.5 (27.1)	89.4 (24.2)	<0.001
VLDL cholesterol (mg/dL)	18.1 (6.7)	17.8 (8.3)	0.66
Total cholesterol/HDL ratio	3.5 (0.5)	3.3 (0.5)	<0.001
Triglycerides	90.7 (33.4)	89.2 (41.3)	0.66

^a^ *t* test

Table 2 shows the sociodemographic characteristics of the sample. Breast feeding less than 4 months was significantly associated with NAFLD. However, family income and parental education were not. We found that the data obtained on dietary habits (using a 3 day recall) and physical activity from the adolescents were unreliable, and these data were therefore not analysed.

**Table 2:** Sociodemographic variables between children with NAFLD and controls

Demographic variable	NAFLD (*N* = 42) Number (%)	Controls (*N* = 373) Number (%)	*p* value
Family income (SLR)			
< 10,000	3 (12.5)	37 (9.9)	
10,000–24,999	14 (33.3)	145 (39.1)	0.58
25,000–49,999	17 (40.5)	146 (39.1)	
> 50,000	8 (19.0)	45 (12.3)	
Maternal Education			
Primary or less	6 (14,3)	42 (10.9)	
Up to O/L	24 (57.1)	248 (66.4)	0.39
Up to A/L	11 (26.2)	66 (17.7)	
Graduates	1(2.4)	17 (4.2)	
Paternal Education			
Primary or less	4 (9.5)	37 (9.9)	
Up to Grade 10	23 (54.8)	249 (66.7)	0.59
Up to Grade 12	11 (26.2)	66 (17.4)	
Graduates	3 (7.1)	21 (5.6)	
Period of Gestation^a^			
< 37 weeks	18 (42.9)	133 (35.5)	0.66
> 37 weeks	23(54.8)	216 (57.9.3)	
Breast feeding^a^			
< 4 months	14 (33.3)	64 (17.1)	
4–5 months	12 (28.6)	182 (48.8)	0.02
5–6 months	10 (23.8)	87 (23.3)	
> 6 months	3 (7.1)	17 (4.6)	
Birth order^a^			
First in the family	20 (47.6)	167 (44.8)	
Second in the family	18 (42.9)	133 (36.2)	0.50
Third or higher	00 (0.0)	58 (15.8)	

^a^ missing data, *SLR* Sri Lankan Rupees

We also analysed the association between components of MetS and NAFLD **(**Table 3 **)**. The odds of having at least one feature of MetS was at least 6.0 times higher among those with NAFLD and this relationship was independent of BMI Z score.(OR among the obese/overweight was 5.6 and among the others 6.2). High waist circumference (prevalence risk ratio 83.3/20.3, 4.1) high triglycerides (prevalence risk ratio 14.3/5.8, 2.5) and a body mass index over 25 (prevalence risk ration 40.5/4.8, 8.4) were significantly associated with NAFLD. None of the adolescents had low HDL cholesterol levels. Although only 1 adolescent had fasting blood sugar over 100 mg/dL, the mean HOMA-IR, was significantly higher in adolescents with NAFLD compared to controls [3.7 (SD 3.8) in NAFLD vs. 1.9 (SD 0.93) in controls, *p* < 0.0001].

**Table 3:** Association between components of metabolic syndrome and NAFLD

Variable	NAFLD Number (%)	Controls Numb er (%)	p value
High waist circumference	35 (83.3)	76 (20.3)	<0.0001^a^
High Triglycerides	6 (14.3)	22 (5.8)	0.033^a^
Low HDL	0	0	–
Fasting blood sugar	1 (2.4)	1 (0.3)	0.056
Body Mass Index (Z score)			
<−2SD	0	74 (19.9)	
- 2 to +1	13 (30.9)	281(75.3)	<0.0001^a^
> + 1	29 (67.4)	24 (6.4)	
High Diasolic Pressure	0	5 (1.3)	0.56
High systolic Pressure	1 (2.4)	7 (1.8)	0.06

^a^Chi-squared test

**Fig. 1:**
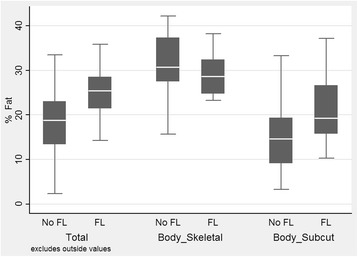
Fat distribution of adolescents with and without NAFLD. For total body fat and subcutaneous fat p < 0.0001, for skeletal fat *p* > 0.05

Table 4 shows the comparisons of the distribution of metabolic derangements in children with and without NAFLD. Distribution of one, two and total number of metabolic derangements were significantly higher among children with NAFLD. Figure 1 shows the distribution of body fat in adolescents with NAFLD and non-NAFLD controls. The total body fat and subcutaneous fat deposits were significantly higher in adolescents with NAFLD (*p* < 0.0001).

**Table 4:** Association between NAFLD and the components of MEtS

Number of metabolic components	With NAFLD No (%)	Without NAFLD No (%)
0	6 (14.3)	283 (75.8)
1	29 (69.1)*	74 (19.8)
2	7 (16.7)*	16 (4.2)

*P < 0.0001 chi squared test

There were no associations between adolescent NAFLD and a family history of components of MetS among family members of the study sample **(**Table 5 **)**.

**Table 5:** Association between medical conditions in the family and NAFLD

Medical Condition	NAFLD Number (%)	Controls Number (%)	*p* value^a^
Diabetes			
Father having diabetes	6 (14.2)	46 (12.3)	0.59
Mother having diabetes	4 (9.5)	25 (6.7)	0.87
Family history of diabetes	10 (23.8)	67 (17.9)	0.32
Myocardial infarction (MI)			
Father having MI	0 (0.0)	10 (2.7)	0.14
Mother having MI	1 (2.3)	6 (1.6)	0.64
Family history of MI	2 (4.8)	18 (4.8)	0.99
Hypertension			
Father having hypertension	8 (19.0)	31 (8.3)	0.12
Mother having hypertension	6 (14.2)	35 (9.3)	0.83
Family history of hypertension	14 (33.3)	66 (17.7)	0.18
Dyslipidaemia			
Father having dyslipidaemia	7 (16.7)	42 (11.3)	0.09
Mother having dyslipidaemia	5 (11.9)	31 (8.1)	0.99
Family history of dyslipidaemia	12 (28.6)	73 (19.6)	0.19

^a^Chi squared test

## Discussion

In this community based study, we found that 8.4% of adolescents living in an urban area in Sri Lanka had NAFLD. Over two thirds of them also had at least one component of MetS. Adolescents with NAFLD had a higher waist circumference, higher BMI, elevated serum triglycerides levels and high amounts of body fat deposition particularly in their subcutaneous tissues. We also found that breast feeding for less than 4 months was significantly associated with NAFLD. Prolonged breast feeding has been shown in previous studies to reduce the prevalence of MetS in children [18]. We did not find an association between adolescent NAFLD and a family history (maternal, paternal or combination) of diseases related to MetS such as diabetes, hypertension, myocardial infarction and dyslipidaemias, in this cohort.

Early studies on the epidemiology of NAFLD in children were based on elevated ALT levels. Two such studies were national health surveys from Korea and the USA, showing a prevalence of 3.6% and 8% respectively [19, 20]. Studies from Japan have demonstrated that the prevalence of NAFLD in children and adolescents range from 2.6 to 4.4% [11]. In China, the prevalence was 1.3% in 7–18 year olds [21]. We found a prevalence of 8.4%. This figure is higher than the 7.6% prevalence rates reported in a meta-analysis of population based studies [4]. The reported prevalence of NAFLD in 17 years old adolescents in the Australian Raine cohort was higher than our fig. (12.8%) [12]. Western life style and dietary patterns could possibly explain the higher prevalence of NAFLD in Australian youth included in that study. The sonographic criteria we used were similar to previous studies. Ayonrinde and co-workers reported that sonographic criteria were much more sensitive than ALT values in detecting NAFLD in community based studies [12]. The reasons for the higher prevalence of NAFLD in our study when compared to prevalence using ALT levels may at least partly be due to this. In addition, the adolescents in our study were from the same geographic community where the prevalence of sonographically defined NAFLD among adults was also found to be high (32.6%) [15].

NAFLD is a disease mainly in affluent societies and the incidence is increasing in most countries [4].The prevalence of NAFLD is positively correlated with gross national income per capita [22]. In our study, family income was not significantly associated with NAFLD. Neither were maternal and paternal education level. This is in contrast to the findings of Zhou et al. who conducted a population based study that included individuals from 7 years to 70 years. In that study, they identified low educational status as a risk factor for developing fatty liver disease [21]. Including adults may have contributed to this difference.

In this study we found that exclusive breast feeding for at least for 4 months has a protective effect on development of NAFLD. Previous studies have also noted the protective effects of breast feeding in developing NAFLD. It had been shown that longer duration of breast feeding and later initiation of supplementary formula milk are associated with a reduction in prevalence of NAFLD. In addition, it was also noted that the prolonged breast feeding reduces the severity of steatosis on sonography. [23]. The authors of that study also found that among adolescents with NAFLD, prolonged breast feeding induces a favourable metabolic profile (low BMI, Low subcutaneous fat, less insulin resistance, low C-reactive protein levels ect..). Furthermore, Nobili et al., in a hospital based study found that odds of complications of NAFLD such as non-alcoholic steatohepatitis and liver fibrosis were less severe in children who were breast fed [24]. Although the exact mechanisms of how breast feeding prevent these complications are far from clear, based on our findings and other studies, we can reiterate the value of prolonged breast feeding in preventing non-communicable diseases including NAFLD.

Studies in adults have shown that approximately two thirds of cases of NAFLD are associated with insulin resistance, and that this figure rises to more than 98% among those with NASH. A strong relationship exists between the number of components of MetS and prevalence of NAFLD, as well as the severity of NAFLD and NASH [14]. Similarly, NAFLD in children is associated with higher fasting glucose, LDL cholesterol, triglycerides, low HDL cholesterol and elevated systolic blood pressure [25]. Assessment of the Raine birth cohort at the age of 17 years found that youth with NAFLD had higher systolic blood pressure, BMI, waist circumference, increased serum triglycerides and low HDL cholesterol [13]. In the current study, we found that high triglyceride levels, a higher waist circumference and a body mass index over 25 Kg/m^2^ were associated with NAFLD. Manco et al. reported that children with obesity and increased waist circumference are at a higher risk of developing advanced NAFLD and hepatic fibrosis [26]. High adiposity trajectories such as BMI, skin fold thickness and mid-arm circumference appear early in life (around 3 years) and tend to influence the severity of NAFLD in later years. [27]. Awareness about these factors may influence development of early preventive measures to curtail the epidemic of NAFLD in Asia.

In one study of biopsy proven NAFLD from the USA, 95% of children had insulin resistance [28]. A community based study in Japanese children also noted an association between NAFLD and insulin resistance [11]. Similarly, we also noted an association between NAFLD and insulin resistance (higher HOMA-IR) though only one adolescent with NAFLD had elevated plasma glucose levels. It is possible that these adolescents need high levels of insulin to maintain normoglycaemia indicating potential to develop non-communicable diseases in early age. A Japanese study comparing metabolic derangements in children with and without NAFLD also found no association between NAFLD and fasting plasma glucose levels. Wickramasinghe et al. investigating metabolic derangement in Sri Lankan children between 5 and 15 years reported that 1.3% had high fasting plasma glucose levels and 23% had low HDL cholesterol [7]. All the adolescents in our sample had normal HDL cholesterol. Despite these differences our results show that adolescents with NAFLD had a higher tendency to have metabolic derangements than controls; the presence of one and two components of the MetS were significantly higher in the NAFLD group, and so was the total number of metabolic derangements.

We found that the total body fat content was higher in adolescents with NAFLD. When the distribution of body fat was analysed, adolescents with NAFLD had a significantly higher percentage of subcutaneous fat and lower percentage of skeletal fat compared to controls. Tominaga and co-workers also illustrated a clear linear relationship between the thickness of subcutaneous fat tissue and presence of NAFLD [17]. The severity of hepatic steatosis was associated with higher subcutaneous adipose tissue thickness in the Raine birth cohort study [13]. A study employing a rapid MRI technique to assess the distribution of body fat in children with NAFLD, found no correlation between the two entities [29]. However, that study had only a small number of children with NAFLD.

The strengths of our study were that it was an adequately powered, community based study, and all participants were of the same age - 14 years (born in the year 2000) at the time of the study. By selecting a group of adolescents of the same age we were able to minimize age related changes in metabolism and fat distribution. Furthermore, fatty liver was diagnosed on established, stringent ultrasound criteria minimising the possibility of over-diagnosis. However, there were several limitations. Magnetic resonance imaging of the liver is considered to be the gold standard for the assessment of fat content in the liver. But it was not practical to perform MRI scans in all the adolescents included in our study. The data on dietary practices and physical activity that were obtained were considered too unreliable for analysis, and inter-observer reliability between the sonographers was not assessed before the study commenced. The data obtained on early feeding practices tend to have recall bias, but we were able to minimize this by checking the child health development records of the adolescents included in the study.

## Conclusions

We have shown that NAFLD is common in Sri Lankan adolescents living in an urban area. The prevalence rate we report is higher than rates in the region and closer to figures reported in Western populations [12, 30, 31]. Adolescent NAFLD was closely associated with early cessation of breast feeding, obesity, increased total and subcutaneous body fat, insulin resistance and several components of the metabolic syndrome. Identifying such modifiable risk factors and encouraging breast feeding for at least 6 months and implementing early community and school based life style modifications may provide preventive and therapeutic windows for NAFLD.

## Abbreviations

ALT: Serum alanine transaminase
HDL: High density lipoproteins
HOMA-IR: Homeostatic modal assessment of insulin resistance
MetS: Metabolic syndrome
MOH: Medical officer of health
NAFLD: Nonalcoholic fatty liver disease
NASH: Nonalcoholic steatohepatitis
TG: Fasting serum triglycerides

## References

[1] Mencin AA, Loomba R, Lavine JE (2015). Caring for children with NAFLD and navigating their care into adulthood. Nat Rev Gastroenterol Hepatol.

[2] Rajindrajith S, Dassanayake A, Hewavisenthi J, de Silva HJ (2008). Advanced hepatic fibrosis and cirrhosis due to non-alcoholic fatty liver disease in Sri Lankan children: a preliminary report. Hepatol Int.

[3] Zimmet P, Alberti G, Kaufman F, Tajima N, Silink M, Arslanian S, Wong G, Bennett P, Shaw J, Caprio S (2007). The metabolic syndrome in children and adolescents. Lancet.

[4] Anderson E, Howe LD, Jones HE, Higgins JP, Lawlor DA, Fraser A (2015). The prevalence of non-alcoholic fatty liver disease in children and adolescents: a systematic review and meta-analysis. PLoS One.

[5] Welsh JA, Karpen S, Vos MB (2013). Increasing prevalence of nonalcoholic fatty liver disease among United States adolescents, 1988-94 to 2007-2010. J Pediatr.

[6] Rodrigues-Colon SM, He F, Bixler EO, Fernandez-Mendoza J, Vgontzas AN, Calhoun S, Zheng ZJ, Liao D (2015). Metabolic syndrome burden in apparently healthy adolescents is associated with cardiac autonomic modulation- Penn state children cohort. Metabolism.

[7] Wickramasinghe VP, Arambepola C, Bandara P, Abeysekera M, Kuruppu S, Dilshan P, Dissanayake BS (2013). Distribution of obesity-related metabolic markers among 5-15 years old children from an urban area of Sri Lanka. Ann Hum Biol.

[8] Kelishadi R, Cook SR, Adibi A, Faghihimani Z, Ghatrehsamani S, Beihaghi A, Salehi H, Khavarian N, Poursafa P (2009). Association of the components of the metabolic syndrome with non-alcoholic fatty liver disease among normal-weight, overweight and obese children and adolescents. Diabetol Metab Syndr.

[9] Pacifico L, Nobilli V, Anania C, Verdecchia P, Chiesa C (2011). Pediatric non- alcoholic fatty liver disease, metabolic syndrome and cardiovascular risk. World J Gastroenterol.

[10] Patton HM, Yates K, Unalp-Arida A, Behling CA, Huang TT, Rosenthal P, Sanyal AJ, Schwimmer JB, Lavine JE (2010). Association between metabolic syndrome and liver histology among children with non-alcoholic fatty liver disease. Am J Gastroenterol.

[11] Tominaga K, Fujimoto E, Suzuki K, Hayashi M, Ichikawa M, Inaba Y (2009). Prevalence of non-alcoholic fatty liver disease in children and relationship to metabolic syndrome, insulin resistance, and waist circumference. Environ Health Prev Med.

[12] Ayonrinde OT, Olynyk JK, Beilin LJ, Mori TA, Pennell CE, de Klerk N, Oddy WH, Shipman P, Adams LA (2011). Gender specific differences in adipose distribution and adipocytokindes influence adolescent nonalcoholic fatty liver disease. Hepatology.

[13] Huang RC, Beilin LJ, Ayonrinde O, Mori TA, Olynyk JK, Burrows S, Hands B, Adams LA (2013). Importance of cardiometabolic risk factors in the association between nonalcoholic fatty liver disease and arterial stiffness in adolescents. Hepatology.

[14] Farrell GC, Wong VW, Chitturi S (2013). NAFLD in Asia- as common and important as in the west. Nat Rev Gastroeterol Hepatol.

[15] Dassanayake AS, Kasturirahne A, Rajindrajith S, Kalubowila U, Chakrawarthi S, de Silva AP, Makaya M, Mizoue T, Kato N, de Silva HJ (2009). Prevalence and risk factors for non-alcoholic fatty liver disease among adults in an urban Sri Lankan population. J Gastroenterol Hepatol.

[16] Saadeh S, Zobair M, Younossi ZM, Remer EM, Gramlich T, Ong JP, Hurley M, Mullan KD, Cooper JN, Sheridan MJ (2002). The utility of radiological imaging in non-alcoholic fatty liver disease. Gastroenterology.

[17] Tominaga K, Kurata JH, Chen YK, Fujimoto E, Miyagawa S, Abe I, Kusano Y (1995). Prevalence of fatty liver in Japanese children and relationship to obesity: an epidemiological ultrasonographic survey. Dig Dis Sci.

[18] Wang J, Zhu Y, Cai L, Jing J, Chen Y, Mai J, Ma L, Ma J (2016). Metabolic syndrome and its associated early-life factors in children and adolescets: a cross sectional study in Guangzhou, China. Public Health Nutr.

[19] Park HS, Han JH, Choi KM, Kim SM (2005). Relation between elevated serum alanine transferase and metabolic syndrome in Korean adolescents. Am J Clin Nutr.

[20] Fraser A, Longnecker MP, Lawlor DA (2007). Prevalence of elevated Alanine Aminotransferase among US adolescents and associated factors: NHANES 1999-2004. Gastroenterology.

[21] Zhou YJ, Li YY, Nie YQ, Ma JX, LG L, Shi SL, Chen MH, Prevalence HPJ (2007). Of fatty liver and its risk factors in the population of south china. World J Gastroenterol.

[22] Zhu JZ, Dai YN, Wang YM, Zhou QY, CH Y, Li YM (2015). Prevalence of non-alcoholic fatty liver disease and economy. Dig Dis Sci.

[23] Ayonrinde OT, Oddy WH, Adams LA, Mori TA, Beilin LJ, de Klerk N, Olynyk JK (2017). Infant nutrition and maternal obesity influence the risk of non-alcoholic fatty liver disease in adolescents. J Hepatology.

[24] Nobilli V, Vedogni G, Alisi A (2009). Pietrobattista am Alterio a, Tiribelli C, Agostoni C. A protective effect of breast feeding on the progression of non-alcoholic fatty liver disease. Arch Dis Child.

[25] Schwimmer JB, Pardee PE, Lavin JE, Blumkin AK, Cook S (2008). Cardiovascular risk factors and the metabolic syndrome in pediatric non-alcoholic fatty liver disease. Criculation.

[26] Manco M, Bedogni G, Marcellini M, Devito R, Ciampalini P, Sartorelli MR, Comparcola D, Piemonte F, Nobili V (2008). Waist circumference correlates with liver fibrosis in children with non-alcoholic steatohepatitis. Gut.

[27] Ayonrinde OT, Olynyk JK, Marsh JA, Beilin LJ, Mori TA, Oddy WH, Adams LA (2015). Childhood adiposity trajectories and risk of nonalcoholic fatty liver disease in adolescents. J Gastroenterol Hepatol.

[28] Schwimmer JB, Deutsch R, Rauch JB, Behling C, Newbury R, Lavine JE (2003). Obesity, insulin resistance, and other clinicopathological correlates of pediatric non-alcoholic fatty liver disease. J Pediatr.

[29] Fishbein MH, Mogren C, Gleason T, Stevens WR (2006). Relationship of hepatic steatosis to adipose tissue distribution in pediatric non-alcoholic fatty liver disease. J Pediatr Gastroenterol Nutr.

[30] Schwimmer JB, Deutsch R, Kahen T, Lavine JE, Stanley C, Behling C (2006). Prevalence of fatty liver in children and adolescents. Pediatrics.

[31] Doycheva I, Watt KD, Alkhouri N (2017). Nonalcoholic fatty liver disease in adolescents and young adults: the next frontier in the epidemic. Hepatology.

